# 2-[(*E*)-2-(4-Eth­oxy­phen­yl)ethen­yl]-1-methyl­pyridinium 4-chloro­benzene­sulfonate monohydrate^1^

**DOI:** 10.1107/S1600536810053572

**Published:** 2011-01-29

**Authors:** Suchada Chantrapromma, Kullapa Chanawanno, Hoong-Kun Fun

**Affiliations:** a Crystal Materials Research Unit, Department of Chemistry, Faculty of Science, Prince of Songkla University, Hat-Yai, Songkhla 90112, Thailand; b X-ray Crystallography Unit, School of Physics, Universiti Sains Malaysia, 11800 USM, Penang, Malaysia

## Abstract

In the title compound, C_16_H_18_NO^+^·C_6_H_4_ClO_3_S^−^·H_2_O, the cation exists in an *E* configuration with respect to the ethenyl bond and is slightly twisted with a dihedral angle of 9.85 (5)° between the pyridinium and the benzene rings. The anion is inclined to the cation with the dihedral angles between the benzene ring of the anion and the pyridinium and benzene rings of the cation of 78.33 (6) and 68.73 (6)°, respectively. In the crystal, the cations and anions are arranged alternately into head-to-head ribbons along the *c* axis, with the cationic ribbons stacked along the *b* axis. The crystal is consolidated by O—H⋯O hydrogen bonds, weak C—H⋯O and C—H⋯π inter­actions. π–π inter­actions with centroid–centroid distances of 3.6111 (7) and 3.6466 (7) Å are also observed.

## Related literature

For background to and the biological activity of quaternary ammonium compounds, see: Armitage *et al.* (1929 ▶); Browning *et al.* (1922 ▶); Chanawanno *et al.* (2010 ▶); Chantrapromma *et al.* (2010 ▶); Wainwright & Kristiansen (2003 ▶). For related structures, see: Fun *et al.* (2010 ▶). For bond-length data, see: Allen *et al.* (1987 ▶). For the stability of the temperature controller used in the data collection, see: Cosier & Glazer (1986 ▶).
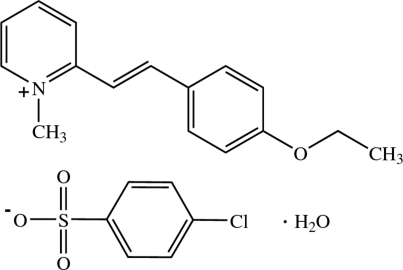


## Experimental

### 

#### Crystal data

C_16_H_18_NO^+^·C_6_H_4_ClO_3_S^−^·H_2_O
*M* *_r_* = 449.94
Monoclinic, 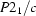

*a* = 9.7568 (5) Å
*b* = 6.5284 (3) Å
*c* = 34.6568 (15) Å
β = 104.784 (1)°
*V* = 2134.43 (17) Å^3^
*Z* = 4
Mo *K*α radiation
μ = 0.31 mm^−1^
*T* = 100 K
0.45 × 0.32 × 0.13 mm

#### Data collection

Bruker APEX DUO CCD area-detector diffractometer
Absorption correction: multi-scan (*SADABS*; Bruker, 2009 ▶) *T*_min_ = 0.873, *T*_max_ = 0.962
30082 measured reflections
7670 independent reflections
6483 reflections with *I* > 2σ(*I*)
*R*_int_ = 0.036

#### Refinement

*R*[*F* ^2^ > 2σ(*F* ^2^)] = 0.040
*wR*(*F* ^2^) = 0.141
*S* = 1.11
7670 reflections
273 parameters
H-atom parameters constrained
Δρ_max_ = 0.54 e Å^−3^
Δρ_min_ = −0.60 e Å^−3^

### 

Data collection: *APEX2* (Bruker, 2009 ▶); cell refinement: *SAINT* (Bruker, 2009 ▶); data reduction: *SAINT*; program(s) used to solve structure: *SHELXTL* (Sheldrick, 2008 ▶); program(s) used to refine structure: *SHELXTL*; molecular graphics: *SHELXTL*; software used to prepare material for publication: *SHELXTL* and *PLATON* (Spek, 2009 ▶).

## Supplementary Material

Crystal structure: contains datablocks global, I. DOI: 10.1107/S1600536810053572/rz2539sup1.cif

Structure factors: contains datablocks I. DOI: 10.1107/S1600536810053572/rz2539Isup2.hkl

Additional supplementary materials:  crystallographic information; 3D view; checkCIF report

## Figures and Tables

**Table 1:** Hydrogen-bond geometry (Å, °) *Cg*2 and *Cg*3 are the centroids of the C8–C13 and C17–C22 rings, respectively.

*D*—H⋯*A*	*D*—H	H⋯*A*	*D*⋯*A*	*D*—H⋯*A*
O1*W*—H1*W*1⋯O4^i^	0.91	1.95	2.8148 (16)	158
O1*W*—H2*W*1⋯O2	0.82	2.11	2.9265 (14)	173
C1—H1*A*⋯O1*W*^ii^	0.93	2.23	3.1544 (17)	176
C2—H2*A*⋯O1*W*^iii^	0.93	2.44	3.2200 (17)	142
C4—H4*A*⋯O2^i^	0.93	2.50	3.3768 (17)	158
C6—H6*A*⋯O3^iv^	0.93	2.56	3.4308 (17)	155
C13—H13*A*⋯O3^iv^	0.93	2.51	3.3859 (17)	157
C16—H16*A*⋯O4^v^	0.96	2.57	3.3766 (18)	142
C16—H16*B*⋯O3^iv^	0.96	2.50	3.1307 (17)	124
C22—H22*A*⋯O4	0.93	2.56	2.9246 (17)	104
C9—H9*A*⋯*Cg*3^i^	0.93	2.90	3.5924 (13)	132
C12—H12*A*⋯*Cg*3^iv^	0.93	2.96	3.7431 (13)	143
C15—H15*C*⋯*Cg*2^vi^	0.96	2.87	3.6918 (14)	145

Symmetry codes: (i) 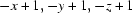; (ii) 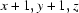; (iii) 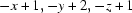; (iv) 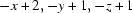; (v) 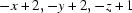; (vi) 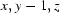.

**Fig. 1.**
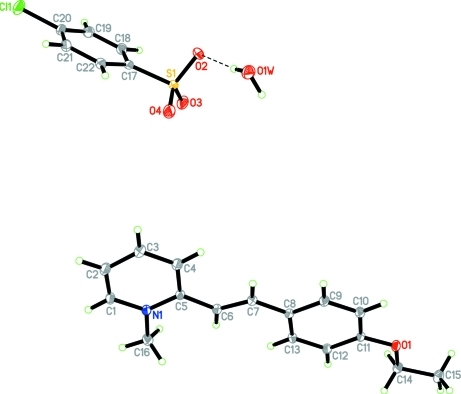
The molecular structure of the title compound, with 50% probability displacement ellipsoids and the atom-numbering scheme. Hydrogen bond was shown as dashed line.

**Fig. 2.**
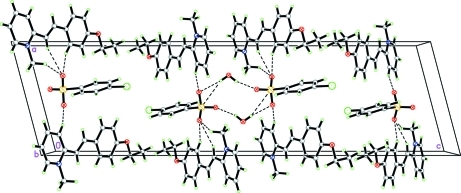
The crystal packing of the title compound viewed down the b axis. Hydrogen bonds were shown as dashed lines.

## supplementary crystallographic information

### Comment

Various quaternary ammonium compounds, such as styryl pyridinium derivatives, exhibit antiseptic properties (Armitage *et al.*, 1929; Browning *et al.*, 1922; Wainwright & Kristiansen, 2003). From our previous investigation on bioactive styryl pyridinium compounds, we found that dimethylaminostyryl pyridinium 4-substituted-benzenesulfonates possess high activity against both susceptible and methicillin-resistant *Staphylococcus aureus* (MRSA) (Chanawanno *et al.*, 2010). In continuing our on-going research on biologically-active quaternary ammonium compounds (Chanawanno *et al.*, 2010; Chantrapromma *et al.*, 2010), the title pyridinium derivative (I) was synthesized. Our results show that (I) is moderately active against the MRSA with the MIC value = 75 µg/ml, whereas it is inactive against susceptible *Staphylococcus aureus*. Herein we report the crystal structure of (I).

In the title compound (Fig. 1), the cation exists in an *E* configuration with respect to the ethenyl bond [torsion angle C5—C6—C7—C8 = 179.53 (11)°]. The cation is slightly twisted with a dihedral angle between the N1/C1–C5 pyridinium and C8–C13 benzene rings of 9.85 (5)°. The ethoxy group is slightly twisted from the mean plane of the attached benzene ring with the torsion angle C11–O1–C14–C15 = -174.84 (10)°. The 4-chlorobenzenesulfonate anion is inclined to the cation as indicated by the dihedral angles between the benzene ring of the anion and the pyridinium and benzene rings of the cation of 78.33 (6) and 68.73 (6)°, respectively. The water molecule forms an O—H···O hydrogen bond with the anion (Table 1). Bond distances in (I) have normal values (Allen *et al.*, 1987) and are comparable to those observed in a related structure (Fun *et al.*, 2010).

In the crystal (Fig. 2), cations and anions are arranged alternatively into head-to-head ribbons along the *c* axis, with the cationic ribbons stacked along the *b* axis. The water molecules are linked to the anions by O—H···O hydrogen bonds and to the cations by C—H···O weak interactions. The crystal is consolidated by O—H···O hydrogen bonds, weak C—H···O and C—H···π interactions (Table 1). π–π interactions with distances Cg_1_···Cg_1_ = 3.6466 (7) Å (symmetry code; 2-x, 2-y, 1-z) and Cg_1_···Cg_2_ = 3.6466 (7) Å (symmetry code; x, 1+y, z) are observed (Cg_1_, Cg_2_ and Cg_3_ are the centroids of N1/C1–C5, C8–C13 and C17–C22 rings, respectively).

### Experimental

The title compound was prepared according to our reported procedure (Chanawanno *et al.*, 2010). Yellow block-shaped single crystal of the title compound suitable for *x*-ray structure determination were recrystallized from methanol by slow evaporation of the solvent at room temperature after a few weeks. M. p. 458-459 K.

### Refinement

All H atoms were positioned geometrically and allowed to ride on their parent atoms, with d(O-H) = 0.82 and 0.91 Å, d(C-H) = 0.93 Å for aromatic and CH and 0.96 Å for CH_3_ atoms. The *U*_iso_ values were constrained to be 1.5*U*_eq_ of the carrier atom for methyl H atoms and 1.2*U*_eq_ for the remaining H atoms. A rotating group model was used for the methyl groups. The highest residual electron density peak is located at 0.60 Å from atom C4 and the deepest hole is located at 0.53 Å from atom S1.

### Crystal data

**Table:**

C_16_H_18_NO^+^·C_6_H_4_ClO_3_S^−^·H_2_O	*F*(000) = 944
*M**_r_* = 449.94	*D*_x_ = 1.400 Mg m^−^^3^
Monoclinic, *P*2_1_/*c*	Melting point = 458–459 K
Hall symbol: -P 2ybc	Mo *K*α radiation, λ = 0.71073 Å
*a* = 9.7568 (5) Å	Cell parameters from 7670 reflections
*b* = 6.5284 (3) Å	θ = 2.7–32.5°
*c* = 34.6568 (15) Å	µ = 0.31 mm^−^^1^
β = 104.784 (1)°	*T* = 100 K
*V* = 2134.43 (17) Å^3^	Block, yellow
*Z* = 4	0.45 × 0.32 × 0.13 mm

### Data collection

**Table:**

Bruker APEX DUO CCD area-detector diffractometer	7670 independent reflections
Radiation source: sealed tube	6483 reflections with *I* > 2σ(*I*)
graphite	*R*_int_ = 0.036
φ and ω scans	θ_max_ = 32.5°, θ_min_ = 2.7°
Absorption correction: multi-scan (*SADABS*; Bruker, 2009)	*h* = −14→14
*T*_min_ = 0.873, *T*_max_ = 0.962	*k* = −9→9
30082 measured reflections	*l* = −52→52

### Refinement

**Table:**

Refinement on *F*^2^	Primary atom site location: structure-invariant direct methods
Least-squares matrix: full	Secondary atom site location: difference Fourier map
*R*[*F*^2^ > 2σ(*F*^2^)] = 0.040	Hydrogen site location: inferred from neighbouring sites
*wR*(*F*^2^) = 0.141	H-atom parameters constrained
*S* = 1.11	*w* = 1/[σ^2^(*F*_o_^2^) + (0.0825*P*)^2^ + 0.5425*P*] where *P* = (*F*_o_^2^ + 2*F*_c_^2^)/3
7670 reflections	(Δ/σ)_max_ = 0.001
273 parameters	Δρ_max_ = 0.54 e Å^−^^3^
0 restraints	Δρ_min_ = −0.60 e Å^−^^3^

### Special details

**Table:**

Experimental. The crystal was placed in the cold stream of an Oxford Cryosystems Cobra open-flow nitrogen cryostat (Cosier & Glazer, 1986) operating at 100.0 (1) K.
Geometry. All esds (except the esd in the dihedral angle between two l.s. planes) are estimated using the full covariance matrix. The cell esds are taken into account individually in the estimation of esds in distances, angles and torsion angles; correlations between esds in cell parameters are only used when they are defined by crystal symmetry. An approximate (isotropic) treatment of cell esds is used for estimating esds involving l.s. planes.
Refinement. Refinement of F^2^ against ALL reflections. The weighted R-factor wR and goodness of fit S are based on F^2^, conventional R-factors R are based on F, with F set to zero for negative F^2^. The threshold expression of F^2^ > 2sigma(F^2^) is used only for calculating R-factors(gt) etc. and is not relevant to the choice of reflections for refinement. R-factors based on F^2^ are statistically about twice as large as those based on F, and R- factors based on ALL data will be even larger.

### Fractional atomic coordinates and isotropic or equivalent isotropic displacement parameters (Å^2^)

**Table:**

	*x*	*y*	*z*	*U*_iso_*/*U*_eq_	
Cl1	0.61088 (4)	1.16478 (6)	0.733973 (10)	0.02817 (10)	
S1	0.57932 (3)	0.55765 (5)	0.591654 (9)	0.01591 (8)	
O1	0.90170 (9)	−0.18011 (14)	0.30285 (3)	0.01455 (17)	
O2	0.44294 (10)	0.45645 (16)	0.58702 (3)	0.02007 (19)	
O3	0.69824 (11)	0.41862 (18)	0.60401 (3)	0.0256 (2)	
O4	0.58242 (13)	0.68270 (18)	0.55696 (3)	0.0281 (2)	
N1	1.05293 (11)	0.97676 (16)	0.44598 (3)	0.01308 (18)	
C1	1.03405 (14)	1.15606 (19)	0.46387 (3)	0.0154 (2)	
H1A	1.1125	1.2364	0.4755	0.018*	
C2	0.90154 (14)	1.2210 (2)	0.46519 (3)	0.0167 (2)	
H2A	0.8895	1.3445	0.4773	0.020*	
C3	0.78515 (14)	1.0976 (2)	0.44795 (4)	0.0172 (2)	
H3A	0.6943	1.1377	0.4486	0.021*	
C4	0.80564 (13)	0.9157 (2)	0.42988 (4)	0.0156 (2)	
H4A	0.7280	0.8333	0.4186	0.019*	
C5	0.94160 (12)	0.85327 (18)	0.42827 (3)	0.0125 (2)	
C6	0.96936 (13)	0.66430 (18)	0.40911 (3)	0.0135 (2)	
H6A	1.0627	0.6205	0.4132	0.016*	
C7	0.86537 (13)	0.55033 (19)	0.38569 (3)	0.0141 (2)	
H7A	0.7730	0.5972	0.3823	0.017*	
C8	0.88342 (12)	0.36117 (18)	0.36522 (3)	0.01223 (19)	
C9	0.76163 (12)	0.2621 (2)	0.34228 (3)	0.0150 (2)	
H9A	0.6729	0.3189	0.3406	0.018*	
C10	0.77130 (12)	0.0822 (2)	0.32213 (4)	0.0148 (2)	
H10A	0.6894	0.0186	0.3073	0.018*	
C11	0.90392 (12)	−0.00427 (18)	0.32398 (3)	0.01176 (19)	
C12	1.02673 (12)	0.09169 (19)	0.34644 (3)	0.0131 (2)	
H12A	1.1153	0.0351	0.3478	0.016*	
C13	1.01532 (12)	0.27234 (19)	0.36666 (3)	0.0131 (2)	
H13A	1.0973	0.3357	0.3815	0.016*	
C14	1.03411 (12)	−0.28231 (19)	0.30496 (3)	0.0143 (2)	
H14A	1.0752	−0.3321	0.3318	0.017*	
H14B	1.1008	−0.1893	0.2977	0.017*	
C15	1.00046 (14)	−0.4587 (2)	0.27591 (4)	0.0183 (2)	
H15A	1.0860	−0.5317	0.2761	0.028*	
H15B	0.9593	−0.4071	0.2496	0.028*	
H15C	0.9347	−0.5495	0.2836	0.028*	
C16	1.20000 (13)	0.9193 (2)	0.44648 (4)	0.0182 (2)	
H16A	1.2635	1.0262	0.4590	0.027*	
H16B	1.2073	0.9000	0.4196	0.027*	
H16C	1.2247	0.7942	0.4612	0.027*	
C17	0.59473 (12)	0.7331 (2)	0.63174 (3)	0.0141 (2)	
C18	0.63847 (13)	0.6617 (2)	0.67086 (4)	0.0172 (2)	
H18A	0.6637	0.5250	0.6757	0.021*	
C19	0.64444 (13)	0.7944 (2)	0.70261 (4)	0.0186 (2)	
H19A	0.6739	0.7480	0.7288	0.022*	
C20	0.60549 (13)	0.9978 (2)	0.69447 (4)	0.0178 (2)	
C21	0.56337 (14)	1.0721 (2)	0.65572 (4)	0.0183 (2)	
H21A	0.5385	1.2090	0.6509	0.022*	
C22	0.55904 (13)	0.9387 (2)	0.62424 (4)	0.0168 (2)	
H22A	0.5323	0.9865	0.5981	0.020*	
O1W	0.28876 (11)	0.44954 (18)	0.50268 (3)	0.0249 (2)	
H1W1	0.3425	0.3848	0.4886	0.037*	
H2W1	0.3382	0.4499	0.5257	0.037*	

### Atomic displacement parameters (Å^2^)

**Table:**

	*U*^11^	*U*^22^	*U*^33^	*U*^12^	*U*^13^	*U*^23^
Cl1	0.03033 (18)	0.0307 (2)	0.02448 (16)	−0.00245 (14)	0.00880 (13)	−0.01471 (13)
S1	0.01448 (13)	0.01772 (15)	0.01687 (14)	−0.00511 (10)	0.00646 (10)	−0.00574 (10)
O1	0.0143 (4)	0.0133 (4)	0.0160 (4)	−0.0007 (3)	0.0037 (3)	−0.0052 (3)
O2	0.0146 (4)	0.0231 (5)	0.0224 (4)	−0.0076 (3)	0.0045 (3)	−0.0059 (4)
O3	0.0161 (4)	0.0260 (5)	0.0345 (5)	0.0022 (4)	0.0063 (4)	−0.0133 (4)
O4	0.0444 (6)	0.0246 (5)	0.0195 (4)	−0.0121 (5)	0.0161 (4)	−0.0056 (4)
N1	0.0164 (4)	0.0110 (4)	0.0123 (4)	−0.0023 (4)	0.0045 (3)	−0.0013 (3)
C1	0.0217 (5)	0.0119 (5)	0.0127 (4)	−0.0028 (4)	0.0049 (4)	−0.0019 (4)
C2	0.0238 (6)	0.0132 (5)	0.0136 (5)	0.0003 (4)	0.0057 (4)	−0.0017 (4)
C3	0.0198 (5)	0.0167 (5)	0.0152 (5)	0.0019 (4)	0.0049 (4)	−0.0024 (4)
C4	0.0158 (5)	0.0155 (5)	0.0157 (5)	−0.0008 (4)	0.0043 (4)	−0.0031 (4)
C5	0.0158 (5)	0.0114 (5)	0.0109 (4)	−0.0016 (4)	0.0044 (4)	−0.0008 (4)
C6	0.0158 (5)	0.0114 (5)	0.0138 (4)	−0.0014 (4)	0.0049 (4)	−0.0018 (4)
C7	0.0153 (5)	0.0135 (5)	0.0146 (5)	−0.0012 (4)	0.0056 (4)	−0.0022 (4)
C8	0.0143 (4)	0.0115 (5)	0.0116 (4)	−0.0016 (4)	0.0045 (3)	−0.0017 (4)
C9	0.0126 (4)	0.0167 (5)	0.0165 (5)	−0.0007 (4)	0.0050 (4)	−0.0044 (4)
C10	0.0122 (4)	0.0164 (5)	0.0156 (5)	−0.0026 (4)	0.0032 (4)	−0.0042 (4)
C11	0.0139 (4)	0.0111 (5)	0.0105 (4)	−0.0013 (4)	0.0036 (3)	−0.0016 (4)
C12	0.0126 (4)	0.0128 (5)	0.0138 (4)	−0.0005 (4)	0.0031 (4)	−0.0018 (4)
C13	0.0133 (4)	0.0124 (5)	0.0132 (4)	−0.0024 (4)	0.0025 (4)	−0.0020 (4)
C14	0.0162 (5)	0.0125 (5)	0.0143 (4)	0.0021 (4)	0.0042 (4)	−0.0008 (4)
C15	0.0225 (6)	0.0149 (5)	0.0172 (5)	0.0032 (4)	0.0043 (4)	−0.0032 (4)
C16	0.0156 (5)	0.0181 (6)	0.0216 (5)	−0.0030 (4)	0.0057 (4)	−0.0040 (4)
C17	0.0114 (4)	0.0163 (5)	0.0153 (5)	−0.0029 (4)	0.0046 (4)	−0.0035 (4)
C18	0.0157 (5)	0.0174 (6)	0.0180 (5)	−0.0009 (4)	0.0032 (4)	−0.0019 (4)
C19	0.0169 (5)	0.0232 (6)	0.0151 (5)	−0.0020 (5)	0.0030 (4)	−0.0022 (4)
C20	0.0149 (5)	0.0206 (6)	0.0184 (5)	−0.0027 (4)	0.0052 (4)	−0.0079 (5)
C21	0.0174 (5)	0.0155 (6)	0.0220 (5)	−0.0015 (4)	0.0051 (4)	−0.0038 (4)
C22	0.0158 (5)	0.0179 (6)	0.0169 (5)	−0.0028 (4)	0.0045 (4)	−0.0015 (4)
O1W	0.0202 (4)	0.0337 (6)	0.0203 (4)	−0.0034 (4)	0.0042 (4)	−0.0082 (4)

### Geometric parameters (Å, °)

**Table:**

Cl1—C20	1.7405 (13)	C10—C11	1.3982 (16)
S1—O3	1.4491 (11)	C10—H10A	0.9300
S1—O2	1.4573 (10)	C11—C12	1.3994 (16)
S1—O4	1.4599 (11)	C12—C13	1.3906 (16)
S1—C17	1.7772 (12)	C12—H12A	0.9300
O1—C11	1.3587 (14)	C13—H13A	0.9300
O1—C14	1.4392 (14)	C14—C15	1.5096 (17)
N1—C1	1.3589 (16)	C14—H14A	0.9700
N1—C5	1.3662 (15)	C14—H14B	0.9700
N1—C16	1.4791 (16)	C15—H15A	0.9600
C1—C2	1.3724 (18)	C15—H15B	0.9600
C1—H1A	0.9300	C15—H15C	0.9600
C2—C3	1.3960 (18)	C16—H16A	0.9600
C2—H2A	0.9300	C16—H16B	0.9600
C3—C4	1.3808 (18)	C16—H16C	0.9600
C3—H3A	0.9300	C17—C18	1.3939 (17)
C4—C5	1.4025 (17)	C17—C22	1.3938 (18)
C4—H4A	0.9300	C18—C19	1.3899 (18)
C5—C6	1.4587 (16)	C18—H18A	0.9300
C6—C7	1.3496 (16)	C19—C20	1.390 (2)
C6—H6A	0.9300	C19—H19A	0.9300
C7—C8	1.4570 (16)	C20—C21	1.3878 (19)
C7—H7A	0.9300	C21—C22	1.3886 (18)
C8—C13	1.4008 (16)	C21—H21A	0.9300
C8—C9	1.4071 (16)	C22—H22A	0.9300
C9—C10	1.3820 (17)	O1W—H1W1	0.9078
C9—H9A	0.9300	O1W—H2W1	0.8195
			
O3—S1—O2	112.85 (7)	C13—C12—C11	119.52 (11)
O3—S1—O4	114.23 (7)	C13—C12—H12A	120.2
O2—S1—O4	111.89 (7)	C11—C12—H12A	120.2
O3—S1—C17	105.73 (6)	C12—C13—C8	121.61 (10)
O2—S1—C17	105.74 (5)	C12—C13—H13A	119.2
O4—S1—C17	105.53 (6)	C8—C13—H13A	119.2
C11—O1—C14	118.27 (9)	O1—C14—C15	106.27 (10)
C1—N1—C5	121.91 (10)	O1—C14—H14A	110.5
C1—N1—C16	117.21 (10)	C15—C14—H14A	110.5
C5—N1—C16	120.88 (10)	O1—C14—H14B	110.5
N1—C1—C2	121.32 (11)	C15—C14—H14B	110.5
N1—C1—H1A	119.3	H14A—C14—H14B	108.7
C2—C1—H1A	119.3	C14—C15—H15A	109.5
C1—C2—C3	118.55 (12)	C14—C15—H15B	109.5
C1—C2—H2A	120.7	H15A—C15—H15B	109.5
C3—C2—H2A	120.7	C14—C15—H15C	109.5
C4—C3—C2	119.62 (12)	H15A—C15—H15C	109.5
C4—C3—H3A	120.2	H15B—C15—H15C	109.5
C2—C3—H3A	120.2	N1—C16—H16A	109.5
C3—C4—C5	121.07 (11)	N1—C16—H16B	109.5
C3—C4—H4A	119.5	H16A—C16—H16B	109.5
C5—C4—H4A	119.5	N1—C16—H16C	109.5
N1—C5—C4	117.53 (11)	H16A—C16—H16C	109.5
N1—C5—C6	119.01 (10)	H16B—C16—H16C	109.5
C4—C5—C6	123.46 (11)	C18—C17—C22	120.23 (11)
C7—C6—C5	122.79 (11)	C18—C17—S1	119.24 (10)
C7—C6—H6A	118.6	C22—C17—S1	120.49 (9)
C5—C6—H6A	118.6	C19—C18—C17	120.14 (12)
C6—C7—C8	126.46 (11)	C19—C18—H18A	119.9
C6—C7—H7A	116.8	C17—C18—H18A	119.9
C8—C7—H7A	116.8	C20—C19—C18	118.72 (12)
C13—C8—C9	117.74 (11)	C20—C19—H19A	120.6
C13—C8—C7	123.90 (10)	C18—C19—H19A	120.6
C9—C8—C7	118.35 (10)	C21—C20—C19	121.92 (12)
C10—C9—C8	121.29 (11)	C21—C20—Cl1	118.95 (11)
C10—C9—H9A	119.4	C19—C20—Cl1	119.13 (10)
C8—C9—H9A	119.4	C20—C21—C22	118.88 (12)
C9—C10—C11	120.12 (11)	C20—C21—H21A	120.6
C9—C10—H10A	119.9	C22—C21—H21A	120.6
C11—C10—H10A	119.9	C21—C22—C17	120.08 (12)
O1—C11—C10	115.42 (10)	C21—C22—H22A	120.0
O1—C11—C12	124.87 (10)	C17—C22—H22A	120.0
C10—C11—C12	119.72 (11)	H1W1—O1W—H2W1	104.3
			
C5—N1—C1—C2	0.44 (17)	O1—C11—C12—C13	179.64 (11)
C16—N1—C1—C2	−179.42 (11)	C10—C11—C12—C13	−0.06 (17)
N1—C1—C2—C3	0.53 (18)	C11—C12—C13—C8	0.17 (18)
C1—C2—C3—C4	−0.49 (18)	C9—C8—C13—C12	−0.45 (17)
C2—C3—C4—C5	−0.49 (19)	C7—C8—C13—C12	−179.79 (11)
C1—N1—C5—C4	−1.39 (16)	C11—O1—C14—C15	−174.84 (10)
C16—N1—C5—C4	178.46 (11)	O3—S1—C17—C18	−38.98 (11)
C1—N1—C5—C6	179.12 (10)	O2—S1—C17—C18	80.92 (11)
C16—N1—C5—C6	−1.03 (16)	O4—S1—C17—C18	−160.37 (10)
C3—C4—C5—N1	1.41 (17)	O3—S1—C17—C22	143.13 (10)
C3—C4—C5—C6	−179.12 (11)	O2—S1—C17—C22	−96.96 (11)
N1—C5—C6—C7	−169.32 (11)	O4—S1—C17—C22	21.75 (11)
C4—C5—C6—C7	11.22 (18)	C22—C17—C18—C19	1.09 (18)
C5—C6—C7—C8	179.53 (11)	S1—C17—C18—C19	−176.80 (9)
C6—C7—C8—C13	−1.26 (19)	C17—C18—C19—C20	0.29 (18)
C6—C7—C8—C9	179.40 (12)	C18—C19—C20—C21	−1.11 (19)
C13—C8—C9—C10	0.64 (18)	C18—C19—C20—Cl1	179.38 (10)
C7—C8—C9—C10	−179.99 (11)	C19—C20—C21—C22	0.52 (19)
C8—C9—C10—C11	−0.54 (19)	Cl1—C20—C21—C22	−179.97 (10)
C14—O1—C11—C10	−177.17 (10)	C20—C21—C22—C17	0.88 (18)
C14—O1—C11—C12	3.12 (17)	C18—C17—C22—C21	−1.69 (18)
C9—C10—C11—O1	−179.49 (11)	S1—C17—C22—C21	176.17 (9)
C9—C10—C11—C12	0.24 (18)		

### Hydrogen-bond geometry (Å, °)

**Table:**

Cg2 and Cg3 are the centroids of the C8–C13 and C17–C22 rings, respectively.

**Table:**

*D*—H···*A*	*D*—H	H···*A*	*D*···*A*	*D*—H···*A*
O1W—H1W1···O4^i^	0.91	1.95	2.8148 (16)	158
O1W—H2W1···O2	0.82	2.11	2.9265 (14)	173
C1—H1A···O1W^ii^	0.93	2.23	3.1544 (17)	176
C2—H2A···O1W^iii^	0.93	2.44	3.2200 (17)	142
C4—H4A···O2^i^	0.93	2.50	3.3768 (17)	158
C6—H6A···O3^iv^	0.93	2.56	3.4308 (17)	155
C13—H13A···O3^iv^	0.93	2.51	3.3859 (17)	157
C16—H16A···O4^v^	0.96	2.57	3.3766 (18)	142
C16—H16B···O3^iv^	0.96	2.50	3.1307 (17)	124
C22—H22A···O4	0.93	2.56	2.9246 (17)	104
C9—H9A···Cg3^i^	0.93	2.90	3.5924 (13)	132
C12—H12A···Cg3^iv^	0.93	2.96	3.7431 (13)	143
C15—H15C···Cg2^vi^	0.96	2.87	3.6918 (14)	145

Symmetry codes: (i) −*x*+1, −*y*+1, −*z*+1;  (ii) *x*+1, *y*+1, *z*; (iii) −*x*+1, −*y*+2, −*z*+1; (iv) −*x*+2, −*y*+1, −*z*+1; (v) −*x*+2, −*y*+2, −*z*+1; (vi) *x*, *y*−1, *z*.
